# The heterogeneous associations between gestational weight gain and adverse pregnancy outcomes in gestational diabetes mellitus according to abnormal glucose metabolism

**DOI:** 10.1038/s41387-023-00239-1

**Published:** 2023-07-04

**Authors:** Qi Wu, Yunyan Chen, Hao Ma, Tao Zhou, Ying Hu, Zhaoxia Liang, Danqing Chen

**Affiliations:** 1 Obstetrical Department, Women’s Hospital, School of Medicine, Zhejiang University, Hangzhou, China; 2 Obstetrical Department, Huzhou Maternity and Child Health Care Hospital, Huzhou, China; 3 Department of Epidemiology, School of Public Health and Tropical Medicine, Tulane University, New Orleans, LA USA

**Keywords:** Risk factors, Weight management

## Abstract

**Objectives:**

The gestational weight gain (GWG) and hyperglycemia are two key factors affecting adverse pregnancy outcomes among women with gestational diabetes mellitus (GDM). We aimed to investigate the combinatorial effect of abnormal glucose metabolism and GWG on adverse outcomes in GDM.

**Methods:**

This retrospective cohort study included 2611 pregnant women with GDM in Women’s Hospital School of Medicine Zhejiang University. Bases on the OGTT glucose levels, we categorized the GDM cohort into three subgroups: impaired fasting glucose (IFG) group, impaired glucose tolerance (IGT) group, and combined impaired glucose (IFG&IGT) group.

**Results:**

Among pregnant women with IGT, insufficient GWG (IGWG) was an independent protective factor for pregnancy-induced hypertension syndrome (PIH) (aOR 0.55, 95% CI 0.32-0.95), macrosomia (0.38, 0.19-0.74) and large for gestational age (0.45, 0.32-0.62), as well as an independent risk factor for low birth weight infants (2.29, 1.24-4.22) and small for gestational age (1.94, 1.17-3.19); and excessive GWG (EGWG) was related to increased risks of PIH (1.68, 1.12-2.52), preterm delivery (1.82, 1.28-2.58), postpartum hemorrhage (1.85, 1.05–3.28), cesarean delivery (1.84, 1.38-2.46) and low body weight infants (2.36, 1.33-4.20). Moreover, EGWG was positively associated with PIH (3.27, 1.09–9.80) in the IFG group. But there were no significant associations between either IGWG or EGWG and any pregnancy outcomes in women with combined IFG&IGT.

**Conclusions:**

The relationships between GWG and adverse outcomes were modified by abnormal glucose metabolism in women with GDM. Our results suggest that more specific GWG recommendations according to their metabolic state are needed for GDM.

## Introduction

Gestational diabetes mellitus (GDM) is defined as an abnormality of glucose metabolism detected for the first time during pregnancy. The incidence of GDM increases gradually with the rise of obesity rate in women of childbearing age, endangering the health of mothers and infants [1, 2]. The gestational weight gain (GWG) and hyperglycemia are two key modifiable factors during pregnancy that contribute to adverse outcomes in GDM [3].

GWG is the reflection of normal pregnancy physiological functions [4]. Several previous studies showed that abnormal GWG was related to higher risks of not only pregnancy complications, but also multiple adverse neonatal outcomes, seriously affecting the life quality of mother and offspring [5–7]. In 2009, the Institute of Medicine (IOM) published recommendations of GWG for singleton pregnancy based on pre-pregnancy body mass index (BMI), which were the most commonly used guidelines [8]. Based on the IOM guidelines, many studies also found in GDM that excessive GWG was an independent risk factor for adverse outcomes [9–11]. However, emerging evidence suggests that the IOM recommendations of GWG may not be applicable to women with a high risk of adverse outcomes, such as obesity, hyperglycemia, hypertension [12].

As another important factor, the glucose levels tested by oral glucose tolerance test (OGTT) varied among pregnant women with GDM because of its specific diagnostic criteria [13, 14]. In the meantime, type of glucose abnormalities in GDM presages different perinatal outcomes [15], partly due to different insulin secretion and insulin sensitivity pattern of impaired fasting glucose (IFG) and impaired glucose tolerance (IGT) [16]. These data suggest that it is necessary to manage GDM according to different abnormal OGTT glucose levels; unfortunately, the evidence is largely lacking.

Actually, there might be a potential association between GWG and hyperglycemia during pregnancy. It is reported that EGWG can aggravate insulin resistance and further increase blood glucose during pregnancy in pregnant women with GDM [17]. Meanwhile, these women with GDM obtain energy from excess fat to raise their glucose levels more easier than normal pregnant women [18]. GWG and hyperglycemia may synergistically affect metabolic function in GDM [19]. Epidemiological studies also showed that the increasing incidence of GDM was parallel to the obesity during pregnancy in recent years [20]. Therefore, we hypothesized that the relationships between GWG and the adverse outcomes might differ according to OGTT glucose levels among women with GDM. To test this hypothesis, we performed a retrospective cohort study among 2611 Chinese women with GDM.

## Subjects and methods

This was a retrospective cohort study conducted in Women’s Hospital School of Medicine Zhejiang University between 1 July 2017 and 30 June 2018. There were 14638 pregnant women giving birth during study period, of which 2639 women with GDM were included in the study. Women with diabetes or hypertension history before pregnancy, multiple pregnancy, stillbirth, fetal anomalies, uncomplete medical records were excluded. We included 2611 women with GDM in the analysis.

GDM was diagnosed according to the recommendations from International Association of Diabetes and Pregnancy Study Groups (IADPSG) using a 75-g OGTT [14]. The criteria of fasting blood glucose (FBG), blood glucose after 1 h (1-h BG) and blood glucose after 2 h (2-h BG) were 5.1 mmol/L (91.8 mg/dL), 10.0 mmol/L (180 mg/dL) and 8.5 mmol/L (153 mg/dL), respectively. Women were diagnosed as GDM when any blood glucose value was greater than the criteria.

The demographic and clinical data, including maternal age, gravidity, parity, height (self-reported at first prenatal care), pre-pregnancy weight (self-reported at first prenatal care), OGTT glucose levels, maternal weight at birth, gestational week at birth, mode of delivery, infant birthweight, pregnancy outcomes, were collected from medical records.

Pre-pregnancy BMI was calculated by dividing pre-pregnancy weight in kilograms by height in meters squared. On the basis of World Health Organization (WHO) criteria, BMI was categorized in 4 groups: underweight (BMI < 18.5 kg/m^2^), normal weight (18.5 ≤ BMI < 25.0 kg/m^2^), overweight (25.0 ≤ BMI < 30.0 kg/m^2^), and obesity (BMI ≥ 30 kg/m^2^).

GWG was derived using the latest maternal weight before delivery minus pre-pregnancy weight. To account for the effect of gestational week at birth, we used the recommendations for weekly GWG instead of total GWG according to IOM 2009 guidelines [8]. Appropriate GWG (AGWG) for the second and third trimester was defined as 0.44-0.58 kg/week in underweight, 0.35-0.50 kg/week in normal weight, 0.23-0.33 kg/week in overweight, and 0.17-0.27 kg/week in obesity. As for the first trimester, calculations assume a 0.5-2 kg weight gain in all BMI groups as an AGWG. Weight gain below and above the IOM targets were categorized as insufficient GWG (IGWG) and excessive GWG (EGWG), respectively.

Pregnancy-induced hypertension syndrome (PIH) included gestational hypertension, preeclampsia or eclampsia, which were diagnosed by recommendations from International Society for the Study of Hypertension in Pregnancy [21]. Preterm delivery was defined as delivery at more than 28 weeks’ gestation but less than 37 weeks’ gestation. Postpartum hemorrhage was defined as more than 500 mL of blood loss after vaginal birth or more than 1000 mL after cesarean delivery. Macrosomia was defined as birthweight equal to or greater than 4000 g, while low body weight infant was less than 2500 g. The infant whose birthweight greater than the 90th percentile or less than 10th percentile for gestational age was diagnosed as large for gestational age (LGA) or small for gestational age (SGA), respectively [22].

To determine whether different blood glucose abnormality in OGTT affected the relationship between GWG and pregnancy outcomes, we divided GDM cohort into three OGTT groups: IFG group (FBG ≥ 5.1 mmol/L [91.8 mg/dL], 1-h BG < 10.0 mmol/L [180 mg/dL] and 2-h BG < 8.5 mmol/L [153 mg/dL], *n* = 234), IGT group (FBG < 5.1 mmol/L [91.8 mg/dL], 1-h BG ≥ 10.0 mmol/L [180 mg/dL] or/and 2-h BG ≥ 8.5 mmol/L [153 mg/dL], *n* = 1970), combined impaired (IFG&IGT) group (FBG ≥ 5.1 mmol/L [91.8 mg/dL], 1-h BG ≥ 10.0 mmol/L [180 mg/dL] or/and 2-h BG ≥ 8.5 mmol/L [153 mg/dL], *n* = 407).

Statistical analyses were performed using SPSS version 20.0. Data were expressed as mean ± standard deviation (SD) or number (percentage). The multiple comparisons of continuous variables were analyzed by ANOVA, and categorical variables were compared using χ2 analyses, as appropriate. Logistic regression analysis was applied to explore the association between GWG category (IGWG, AGWG, EGWG, whereby AGWG was the reference category) and pregnancy outcomes (cesarean delivery, PIH, preterm birth, macrosomia, low body weight infant, LGA, SGA) in three OGTT groups. The odds ratio (OR) with 95% confidence interval (CI) were generally adjusted for maternal age, gravidity, parity, pre-pregnancy BMI, OGTT glucose levels (FBG, 1-h BG, 2-h BG) in multivariable analyses. Power analysis was conducted by Gpower 3.1 and showed sufficient power (å 80%) of our study to detect differences in the results. Statistical significance was considered as *P* < 0.05.

This study was approved by the hospital’s ethics committee (IRB-20200243-R), and informed consent was not required because of using anonymized patient records.

## Results

There were 2611 women with a singleton GDM pregnancy met the study inclusion criteria. The mean maternal age was 32.6 ± 4.6 years. The mean pre-pregnancy BMI and GWG regardless of BMI categories were 21.9 ± 3.1 kg/m^2^ and 12.4 ± 4.1 kg, respectively. Overall, 1417 (54.3%) pregnant women with GDM gained appropriate weight according to IOM weekly recommendations, whereas the rate of IGWG and EGWG were 578 (22.1%) and 616 (23.6%). However, the proportion of GWG categories was different according to guidelines for total weight gain during pregnancy regardless of gestational week. There were only 1152 (44.1%) and 462 (17.7%) women within and above the guidelines, while 997 (38.2%) women had insufficient weight gain during the pregnancy, which indicated that IGWG population will be overestimated if the effect of gestational week of delivery was ignored and therefore affected its relationship with adverse pregnancy outcomes. Furthermore, the average FBG, 1-h BG, and 2-h BG of total were 4.7 ± 0.6 (84.6 ± 10.8), 10.1 ± 1.4 (181.8 ± 25.2), and 8.9 ± 1.4 (160.2 ± 25.2) mmol/L (mg/dL), respectively. (Table 1).

**Table 1:** Maternal characteristics grouped by OGTT glucose levels.

	Total (*n* = 2611)	OGTT groups			
Characteristic		IFG (*n* = 234)	IGT (*n* = 1970)	IFG& IGT (*n* = 407)	*P*
Maternal age (years)	32.6 ± 4.6	31.3 ± 4.6	32.53 ± 4.5*	33.4 ± 4.8*^,#^	<0.001
Gravidity	2.3 ± 1.3	2.4 ± 1.4	2.3 ± 1.3	2.5 ± 1.3^#^	0.004
Parity					0.068
Nulliparous	1266 (48.5)	121 (51.7)	968 (49.1)	177 (43.5)	
Multiparous	1345 (51.5)	113 (48.3)	1002 (50.9)	230 (56.5)	
Prior cesarean delivery	635 (24.3)	46 (19.7)	470 (23.9)	119 (29.2)*	0.015
BMI (kg/m^2^)	21.9 ± 3.1	22.8 ± 3.1	21.5 ± 3.0*	23.4 ± 3.5^#^	<0.001
Underweight	309 (11.8)	13 (5.6)	270 (13.7)*	26 (6.4)*^,#^	<0.001
Normal weight	1926 (73.8)	180 (76.9)	1481 (75.2)*	265 (65.1)*^,#^	
Overweight	333 (12.8)	34 (14.5)	203 (10.3)*	96 (23.6)*^,#^	
Obesity	43 (1.6)	7 (3.0)	16 (0.8)*	20 (4.9)*^,#^	
GWG (kg)	12.4 ± 4.1	12.6 ± 4.8	12.3 ± 4.0	12.7 ± 4.1	0.242
Appropriate	1417 (54.3)	116 (49.6)	1101 (55.9)	200 (49.1)^#^	<0.001
Insufficient	578 (22.1)	55 (23.5)	453 (23.0)	70 (17.2)^#^	
Excessive	616 (23.6)	63 (26.9)	416 (21.1)	137 (33.7)^#^	
Gestational week	38.1 ± 1.8	38.2 ± 2.1	38.2 ± 1.8	37.8 ± 1.9*^,#^	<0.001
OGTT (mmol/L)					
FBG	4.7 ± 0.6	5.4 ± 0.4	4.5 ± 0.3*	5.6 ± 0.6*^,#^	<0.001
1-h BG	10.1 ± 1.4	8.4 ± 1.1	10.1 ± 1.2*	11.2 ± 1.6*^,#^	<0.001
2-h BG	8.9 ± 1.4	7.2 ± 0.9	8.9 ± 1.2*	9.6 ± 1.8*^,#^	<0.001

Data were expressed as mean ± SD or number (percentage). *IFG* impaired fasting glucose group, *IGT* impaired glucose tolerance group, *IFG&IGT* both impaired group, *BMI* body mass index, *GWG* gestational weight gain, *OGTT* oral glucose tolerance test, *FBG* fasting blood glucose, *1-h BG* blood glucose after 1 h, *2-h BG* blood glucose after 2 h. *P < 0.017 vs IFG; ^#^*P* < 0.017 vs IGT.

Based on OGTT glucose levels, 234 (9.0%) women were categorized into the IFG group, 1970 (75.5%) into the IGT group and 407 (15.6%) into the IFG&IGT group. Of these, the IFG group and the IGT group had the lowest post-OGTT glucose and FBG respectively, while the highest OGTT glucose levels were found in the IFG&IGT group (all *P* < 0.001). Among the three OGTT groups, there were significant differences in maternal age, gravidity, pre-pregnancy BMI, GWG, gestational week at birth and prior cesarean delivery (*P* ≤ 0.015 for all comparisons). Women with combined IFG&IGT had the highest maternal age but the lowest gestational week at birth, while women with IGT had the lowest BMI and women with IFG were youngest (*P* < 0.017, compared with other two OGTT groups). In addition, women who gained insufficient weight were more likely to have IFG, while women with EGWG were more likely to have combined IFG and IGT in GDM. (Table 1).

The relationship of OGTT glucose levels and pregnancy outcomes was displayed by Table 2. In regard to maternal outcomes, the prevalence of PIH, preterm delivery and cesarean delivery were associated with different OGTT glucose levels in women with GDM (*P* ≤ 0.001 for all comparisons). Similar results were also found in neonatal outcomes (birth weight, macrosomia, LGA, *P* < 0.001). Interestingly, pregnant women with combined IFG and IGT had significantly increased incidences of PIH (15.7% vs 7.6%), preterm delivery (18.2% vs 11.3%), cesarean delivery (62.9% vs 52.1%), birth weight (3357.7 ± 608.3 g vs 3233.5 ± 515.6 g), macrosomia (13.0% vs 5.2%), LGA (32.7% vs 20.4%), only when compared to women with isolated IGT (all *P* < 0.001). However, as for postpartum hemorrhage, low body weight infants and SGA, there were not significantly different in the three OGTT groups.

**Table 2:** Maternal and neonatal outcomes grouped by OGTT glucose levels.

	Total (*n* = 2611)	OGTT groups			
Outcomes		IFG (*n* = 234)	IGT (*n* = 1970)	IFG& IGT (*n* = 407)	*P*
PIH	233 (8.9)	20 (8.5)	149 (7.6)	64 (15.7)*^,#^	<0.001
Preterm delivery	323 (12.4)	26 (11.1)	223 (11.3)	74 (18.2)^#^	0.001
Cesarean delivery	1409 (54.0)	126 (53.8)	1027 (52.1)	256 (62.9)^#^	<0.001
Postpartum hemorrhage	99 (3.8)	14 (6.0)	67 (3.4)	18 (4.4)	0.144
Birth weight (g)	3259.9 ± 540.2	3311.9 ± 593.4	3233.5 ± 515.6	3357.7 ± 608.3^#^	<0.001
Macrosomia	174 (6.7)	19 (8.1)	102 (5.2)	53 (13.0)^#^	<0.001
Low body weight infants	192 (7.4)	20 (8.5)	141 (7.2)	31 (7.6)	0.725
LGA	595 (22.8)	61 (26.1)	401 (20.4)	133 (32.7)^#^	<0.001
SGA	102 (3.9)	8 (3.4)	85 (4.3)	9 (2.2)	0.126

Data were expressed as mean ± SD or number (percentage). *OGTT* oral glucose tolerance test, *IFG* impaired fasting glucose group, *IGT* impaired glucose tolerance group, *IFG&IGT* both impaired group, PIH pregnancy-induced hypertension syndrome, *LGA* large for gestational age, *SGA* small for gestational age. *P < 0.017 vs IFG; ^#^*P* < 0.017 vs IGT.

Table 3 showed the association of maternal and neonatal outcomes with GWG in pregnant women with GDM, which was adjusted for OGTT groups. Compared to women with AGWG, IGWG was positively associated with low body weight infants (adjusted OR 2.18, 95% CI 1.28-3.72, *P* = 0.004) and SGA (adjusted OR 1.96, 95% CI 1.24-3.11, *P* = 0.004), and negatively associated with macrosomia (adjusted OR 0.41, 95% CI 0.24-0.70, *P* = 0.001) and LGA (adjusted OR 0.49, 95% CI 0.37-0.64, *P* < 0.001). EGWG was an independent risk factor for PIH (adjusted OR 1.78, 95% CI 1.29-2.45, *P* < 0.001), preterm delivery (adjusted OR 1.82, 95% CI 1.37–2.41, *P* < 0.001), cesarean delivery (adjusted OR 1.69, 95% CI 1.33-2.14, *P* < 0.001) and low body weight infants (adjusted OR 1.82, 95% CI 1.10-3.02, *P* = 0.020).

**Table 3:** Association of maternal and neonatal outcomes with GWG in GDM.

Outcomes*	IGWG (*n* = 578)		AGWG (*n* = 1417)	EGWG (*n* = 616)	
	OR (95% CI), *P*	aOR (95% CI), *P*	OR (95% CI), *P*	OR (95% CI), *P*	aOR (95% CI), *P*
PIH	0.74 (0.49-1.12) 0.152	0.71 (0.46-1.09) 0.112	Reference	**2.56 (1.90-3.43)** **<0.001**	**1.78 (1.29-2.45)** **<0.001**
Preterm delivery^†^	1.23 (0.90-1.67) 0.190	1.22 (0.89-1.66) 0.222	Reference	**2.03 (1.56-2.66)** **<0.001**	**1.82 (1.37–2.41)** **<0.001**
Cesarean delivery^‡^	0.89 (0.73-1.08) 0.229	0.86 (0.68-1.10) 0.233	Reference	**1.70 (1.40-2.07)** **<0.001**	**1.69 (1.33-2.14)** **<0.001**
for nulliparous^§^	0.76 (0.57-1.01) 0.060	0.80 (0.59-1.09) 0.160	Reference	**2.25 (1.70-2.96)** **<0.001**	**1.93 (1.43-2.62)** **<0.001**
for multiparous^‡^	1.06 (0.80-1.40) 0.705	0.99 (0.66-1.48) 0.960	Reference	**1.34 (1.01-1.78)** **0.042**	1.36 (0.92-2.01) 0.119
Postpartum hemorrhage	0.86 (0.49-1.52) 0.611	0.88 (0.50-1.55) 0.665	Reference	**1.67 (1.06-2.61)** **0.026**	1.48 (0.93-2.36) 0.100
Macrosomia^||^	**0.45 (0.27-0.76)** **0.002**	**0.41 (0.24-0.70)** **0.001**	Reference	**1.58 (1.13-2.20)** **0.008**	1.35 (0.94-1.96) 0.109
Low body weight infants^||^	**1.81 (1.25-2.62)** **0.002**	**2.18 (1.28-3.72)** **0.004**	Reference	**2.36 (1.67-3.33)** **<0.001**	**1.82 (1.10-3.02)** **0.020**
LGA^¶^	**0.48 (0.37-0.63)** **<0.001**	**0.49 (0.37-0.64)** **<0.001**	Reference	**1.39 (1.12-1.71)** **0.002**	1.17 (0.93-1.47) 0.172
SGA^¶^	**2.01 (1.28-3.17)** **0.003**	**1.96 (1.24-3.11)** **0.004**	Reference	1.21 (0.72-2.02) 0.466	1.27 (0.75-2.17) 0.372

*OR* odds ratio, *aOR* adjusted odds ratio, *IGWG* inadequate gestational weight gain, *AGWG* appropriate gestational weight gain, *EGWG* excessive gestational weight gain, *PIH* pregnancy-induced hypertension syndrome, *LGA* large for gestational age, *SGA* small for gestational age.

^*^All analyses were adjusted for maternal age, gravidity, parity, pre-pregnancy BMI, OGTT glucose levels (FBG, 1-h BG, 2-h BG), and OGTT groups.

^†^The analysis of preterm delivery was extra adjusted for PIH and premature rupture of membranes.

^‡^The analysis of cesarean delivery was extra adjusted for PIH, macrosomia, premature rupture of membranes and prior cesarean delivery, as was the analysis of cesarean delivery for multiparous women, which was restricted to multiparous women only.

^§^The analysis of cesarean delivery for nulliparous women was restricted to nulliparous women only and extra adjusted for PIH, macrosomia and premature rupture of membranes.

^||^The analyses of macrosomia and low body weight infants were extra adjusted for PIH and gestational week.

^¶^The analyses of LGA and SGA were extra adjusted for PIH.

**Fig. 1:**
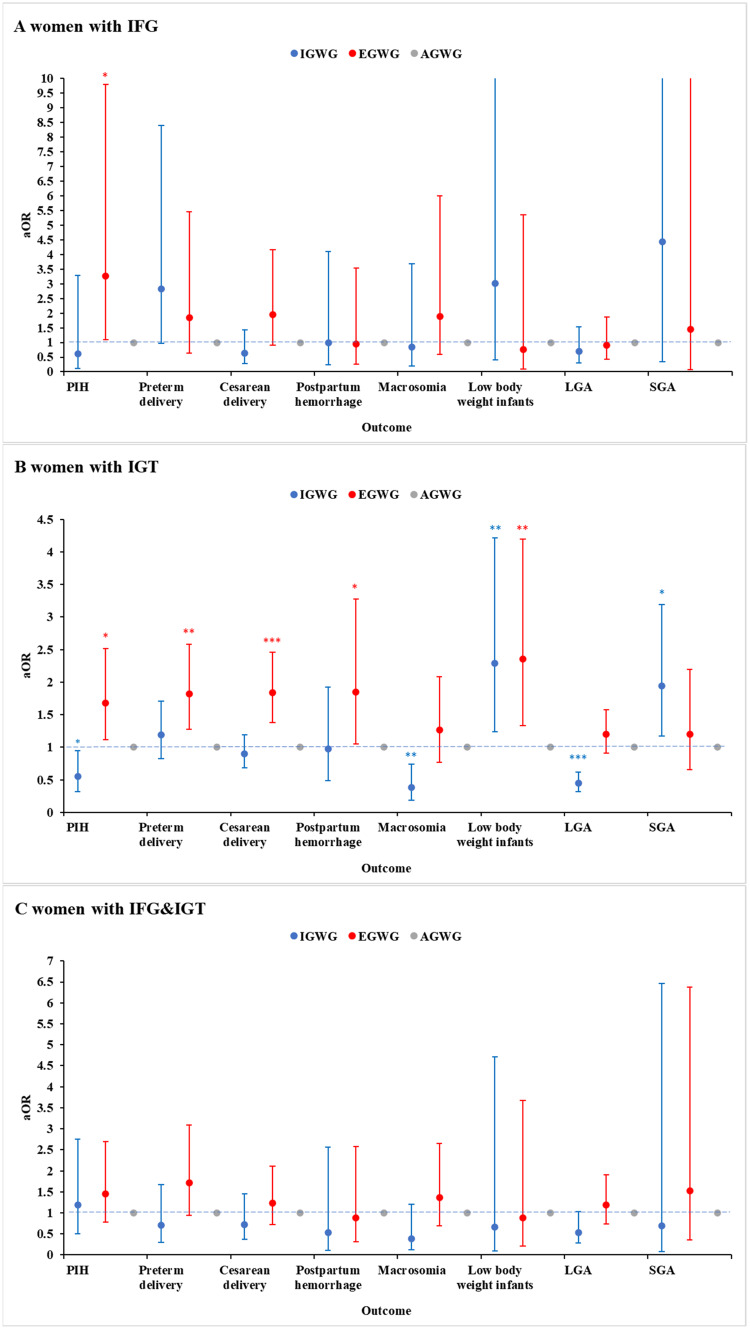
Maternal and neonatal outcomes associated with GWG in GDM women with different abnormal glucose metabolism. (A) IFG. (B) IGT. (C) IFG&IGT. All analyses were adjusted for maternal age, gravidity, parity, pre-pregnancy BMI, OGTT results (FBG, 1 h BG, 2 h BG). The analysis of preterm delivery was extra adjusted for PIH and premature rupture of membranes. The analysis of cesarean delivery was extra adjusted for PIH, macrosomia, premature rupture of membranes and prior cesarean delivery. The analyses of macrosomia and low body weight infants were extra adjusted for PIH and gestational week. The analyses of LGA and SGA were extra adjusted for PIH. Women who had an appropriate weight gain during pregnancy were the reference group. Error bars presented 95% CI. aOR adjusted odds ratio. *P < 0.05, **P < 0.01, ***P < 0.001. IFG impaired fasting glucose group, IGT impaired glucose tolerance group, IFG&IGT, both impaired group; aOR adjusted odds ratio, IGWG inadequate gestational weight gain, AGWG appropriate gestational weight gain, EGWG excessive gestational weight gain, PIH pregnancy-induced hypertension syndrome, LGA large for gestational age, SGA small for gestational age.

To clarify the combinatorial effect of abnormal glucose metabolism and GWG on adverse outcomes in pregnant women with GDM, we used stratified analysis and found the associations between GWG and the pregnancy outcomes were most pronounced in the IGT group. For those women, IGWG was significantly related to neonatal birth weight. Significant positive association was found between IGWG and low body weight infants (adjusted OR 2.29, 95% CI 1.24-4.22, *P* = 0.008) or SGA (adjusted OR 1.94, 95% CI 1.17-3.19, *P* = 0.010), while negative association with macrosomia (adjusted OR 0.38, 95% CI 0.19-0.74, *P* = 0.004) or LGA (adjusted OR 0.45, 95% CI 0.32-0.62, *P* < 0.001). Moreover, the adjusted OR of PIH was 0.55 (95% CI, 0.32-0.95) for women with IGWG compared to those with AGWG in the IGT group. In contrast, pregnant women with IGT who gained excessive weight experienced significantly higher risk of PIH (adjusted OR 1.68, 95% CI 1.12-2.52, *P* = 0.012), preterm delivery (adjusted OR 1.82, 95% CI 1.28-2.58, *P* = 0.001), postpartum hemorrhage (adjusted OR 1.85, 95% CI 1.05-3.28, *P* = 0.035), cesarean delivery for nulliparous (adjusted OR 2.46, 95% CI 1.69-3.57, *P* < 0.001), and low body weight infants (adjusted OR 2.36, 95% CI 1.33-4.20, *P* = 0.003). Furthermore, we only found EGWG was an independent risk factor for PIH in the IFG group (adjusted OR 3.27, 95% CI 1.09-9.80, *P* = 0.034). There was no longer significantly association between GWG and any pregnancy outcomes in adjusted analyses of women with combined IFG&IGT, although EGWG seem to increase the risk of PIH and preterm delivery. (Fig. 1).

In further stratified analysis of GWG, we also found combined IFG&IGT increased the risk of macrosomia in women with AGWG (adjusted OR 2.47, 95% CI 1.05-5.80, *P* = 0.038) and PIH in women with IGWG (adjusted OR 6.61, 95% CI 1.12-38.93, *P* = 0.037); while decreased the risk of low body weight infants in women with IGWG (adjusted OR 0.12, 95% CI 0.02-0.97, *P* = 0.046).

## Discussion

In this study, we for the first time investigated whether different blood glucose abnormality in OGTT modified the association between GWG and pregnancy outcomes in GDM. Significant heterogeneity was observed in relationships between GWG and the adverse maternal and neonatal outcomes in GDM women with IFG, IGT or IFG&IGT.

We found PIH was most closely related to weight gain during pregnancy. Regardless of abnormal glucose metabolism, EGWG increased the risk of PIH, especially among women with isolated IFG. Meanwhile, IGWG was found to be an independently protective factor for PIH only in women with IGT. Similarly, a number of studies generally showed that pregnant women with GDM who gained excessive weight during pregnancy were at a higher risk of PIH, while insufficient weight gain was associated with decreased likelihood of hypertensive disorders [23, 24]. But the associations between preterm delivery and GWG remain controversial in previous studies. A meta-analysis illustrated that preterm delivery in women with GWG above the IOM recommendations showed increased risk [25]. Johnson, J. et al. reported that gaining less weight during pregnancy increased the risk of spontaneous preterm delivery [26]. Pre-pregnancy BMI and subtype of preterm delivery might be factors influencing the different roles of GWG in the assessment [27]. In our stratified analysis with adjustment for confounding factors such as pre-pregnancy BMI, we found that abnormal glucose metabolism might also be an important factor modifying the association between preterm delivery and GWG, that pregnant women with IGT were more likely to have preterm delivery if they gained excessive weight during pregnancy. Furthermore, our findings also demonstrated that women with EGWG in the IGT group had an increased likelihood of cesarean delivery and postpartum hemorrhage compared with those with AGWG, consistent with previous studies [28, 29]. However, there were no relationship between GWG and those adverse pregnancy outcomes for women with IFG in GDM (including isolated IFG and combined IFG&IGT). Generally, GWG was found to be associated with the adverse maternal outcomes only in pregnant women with isolated IGT, while other factors (such as maternal age, pre-pregnancy BMI) might be responsible for adverse pregnancy outcomes in women with IFG.

GDM has been related to birth weight of newborns. Macrosomia and LGA were recognized as common complications in pregnant women with GDM [30, 31]. However, other studies also showed that the incidence of LGA was comparable to that of SGA in intervened GDM group [32, 33]. The IADPSG-HAPO study reported that there was a 30% and 60% increased odds ratio for LGA and SGA in women with GDM [34], whose difference might be greatly related to GWG. It was observed that EGWG was associated with a 1.59-fold increased risk of macrosomia and a 1.40-fold increased risk of LGA in GDM; meanwhile, IGWG increased the risk of low birth weight and SGA [35]. Yi-Ling Chiou et al. also found GWG was significantly associated with perinatal outcomes in both GDM themselves and their newborns [36]. But in our study, joint correlation of abnormal glucose metabolism and GWG with birth weight of newborns was also found in GDM. Similar to the maternal outcomes, GWG was also associated with neonatal weight only in women with IGT, who was most common in this GDM population. Among them, IGWG was a protective factor for macrosomia, LGA and a risk factor for low body weight infants, SGA. Interestingly, EGWG was only associated with an increased risk of low birth weight infants by 2.36 times, but was not associated with the risk of macrosomia and LGA, conflicting with several previous studies. Exploring the causes, abnormal glucose metabolism rather than GWG might play a more important role in macrosomia and LGA for GDM. The results showed that FPG was an independent risk factor for macrosomia (adjusted OR 2.17, 95% CI 1.05-4.47, *P* = 0.036) and LGA (adjusted OR 1.69, 95% CI 1.16-2.47, *P* = 0.007). Similar reasons might exist for insignificant results in the IFG and IFG&IGT groups.

Actually, pathogenesis underlying IFG and IGT are different in general population. Although the insulin sensitivity in IFG and IGT are consistently decreased compared with normal glucose tolerance, their insulin resistance sites are different: IFG is mainly manifested as hepatic insulin resistance with poor suppression of hepatic glucose output, whereas its muscle insulin sensitivity is tend to be normal [37]. The hepatic insulin sensitivity is normal or slightly reduced, but moderate to severe muscle insulin resistance exists in IGT [38]. Besides, they also differ in the pattern of insulin secretion: IFG is more likely to have a decrease in early insulin secretion, which combined with hepatic insulin resistance, causes excessive hepatic glucose production and lead to fasting hyperglycemia. IGT, on the other hand, shows more severe β-cell dysfunction with significantly reduced glucose-induced insulin secretion in both early and late stages, results in post-OGTT hyperglycemia [16, 39]. Considering the differences in the physiological basis of impaired fasting glucose and impaired glucose tolerance, and the similarity in pathogenesis between GDM and type 2 diabetes, the heterogeneity of perinatal outcome among GDM women with different OGTT blood glucose abnormalities is partly predictable. Previous study demonstrated that LGA and shoulder dystocia might be strongly associated with IFG in pregnant women with GDM, while preterm delivery and gestational hypertension appeared to be more closely associated with IGT [40]. Disse et al. found significantly positive relationship between LGA and IFG, and fasting glucose could be highly predictive of LGA delivery [15]. Interestingly, meta-analysis showed linear associations of glucose with perinatal outcomes, and associations of adverse outcomes were stronger for fasting glucose level than for post-load glucose level [20]. In addition, several studies have found that gestational hyperglycemia is related to offspring glucose metabolism and obesity, and the association is stronger in those with more abnormal OGTT values [41, 42]. These results are similar to those of our study, indicating why no significant correlation between GWG and adverse outcomes was found in combined IFG&IGT group, in which abnormal blood glucose might play a more important role.

Furthermore, the interrelationship between hyperglycemia and obesity during pregnancy is also noteworthy [19]. A study reported that EGWG was related to excessive body fat accumulation in GDM, which impaired the function of pancreatic β-cells and result in increased insulin resistance, thus exacerbating hyperglycemia during pregnancy [17]. Compared with pregnant women with normal glucose metabolism, women with GDM are more likely to obtain energy from fat, and their glucose levels will increase significantly with fat intake [18]. In fact, maternal weight is a major concern for GDM. The Hyperglycemia and Adverse Pregnancy Outcome Study found that both GDM and obesity were independently related to the adverse outcomes, and their combination had a greater impact [43]. Our previous study also found that glycosylated hemoglobin level and GWG can jointly affect the occurrence of adverse pregnancy outcomes in GDM [44]. EGWG and hyperglycemia during pregnancy both affect the growth and development of the fetus in utero, leading to abnormal fat deposition and obesity in offspring [45]. These results indicated that GWG and hyperglycemia might synergistically affect metabolic function in GDM, which increased the risk of adverse maternal and neonatal outcomes.

In response to the increasing incidence of GDM globally, effective prevention and treatment for GDM is a topical issue in women of childbearing age. “Precision medicine”, which means the ability to stratify patients by their susceptibility to a particular disease that can be treated in a better way, has become very popular in recent years [46]. In fact, our study indicated that the incidence of inappropriate weight gain during pregnancy and adverse perinatal outcomes were different according to OGTT blood glucose levels of pregnant women with GDM. The adverse outcomes were more likely to occur in pregnant women with IFG& IGT than in pregnant women with IGT. Women with EGWG accounted for 21.1% in the IGT group, while 33.7% in the IFG&IGT group. Similar results were found on BMI that the proportion of overweight and obese women was the lowest in the IGT group and the highest in the IFG&IGT group. The differential results of IFG and IGT in GDM suggested the importance to focus on different aspects for GDM with different abnormal OGTT glucose levels to reduce multiple adverse pregnancy outcomes. These pregnant women with GDM might require more rigorous and specific standards of GWG to balance competing maternal and neonatal risks. Precision treatment is urgently needed in the clinical treatment and intervention of GDM according to metabolic status.

To the best of our knowledge, this was the first study to explore the relationship between GWG and adverse pregnancy outcomes varied by OGTT glucose levels in pregnant women with GDM. Our findings suggest that different recommendations for weight gain during pregnancy need to be provided for GDM with different blood glucose impairment, consistent with the principle of precision medicine. Especially for pregnant women with IGT, proper weight gain during pregnancy is particularly important, while pregnant women with IFG and IFG&IGT should not ignore other potential risk factors (including pre-pregnancy BMI, blood glucose values and so on) when paying attention to GWG. As such the personalized treatment plans can be provided for women with GDM to improve their perinatal outcomes. In addition, gestational week of delivery, a key determinant affecting the adequacy of GWG, was taken into account in our study. We used weekly GWG recommendations rather than total GWG recommendations of IOM to prevented misestimating the actual GWG classification, which might affect its relationship with adverse pregnancy outcomes.

However, there were still several potential limitations in our study. First, this was a hospital-based retrospective study and the sample size was relatively small, especially for women with impaired fasting glucose, which might affect the power and generality of our study. Second, data on type of therapy and glycemic controls during pregnancy after diagnosis of GDM were lacking in this study, which might also affect the risk of adverse outcomes. Large prospective studies are needed in the future to further explore the association between GWG and perinatal outcome in GDM according to metabolic status, and to further explore the role of GDM treatment in it. Third, limited by the unclear pathogenesis of GDM, we cannot make a comprehensive explanation of the different perinatal outcomes caused by different abnormal glucose metabolism in pregnant women with GDM, which is also the direction of our future research. Another limitation was that pre-pregnancy weight was self-reported, therefore reporting bias could not be excluded. But high correlations were found between self-reported and measured weight before pregnancy [47], so it would not seriously affect our conclusions.

In summary, the heterogeneous relationships between GWG and adverse outcomes were found in GDM with different abnormal glucose metabolism. Our findings indicated that pregnant women with GDM might need more specific and strict recommendations to manage weight gain during pregnancy, especially in women with IGT. Stratified management and precision intervention for GDM will be a direction to improve the perinatal outcome of GDM and delaying the occurrence of long-term maternal and infant diseases.

## Data Availability

The data that support the findings of this study are available from the corresponding author [Z.X.L.] upon reasonable request.
